# Phage Cocktail Development against *Aeromonas salmonicida* subsp. *salmonicida* Strains Is Compromised by a Prophage

**DOI:** 10.3390/v13112241

**Published:** 2021-11-08

**Authors:** Nava Hosseini, Valérie E. Paquet, Mahdi Chehreghani, Sylvain Moineau, Steve J. Charette

**Affiliations:** 1 Institut de Biologie Intégrative et des Systèmes (IBIS), Pavillon Charles-Eugène-Marchand, Université Laval, Quebec City, QC G1V 0A6, Canada; nava.hosseini.1@ulaval.ca (N.H.); Valerie.Paquet@criucpq.ulaval.ca (V.E.P.); Sylvain.Moineau@bcm.ulaval.ca (S.M.); 2 Département de Biochimie, de Microbiologie et de Bio-Informatique, Faculté des Sciences et de Génie, Université Laval, Quebec City, QC G1V 0A6, Canada; 3 Centre de Recherche de l’Institut Universitaire de Cardiologie et de Pneumologie de Québec (IUCPQ), Quebec City, QC G1V 4G5, Canada; 4 Department of Mechanical Engineering, McGill University, Montreal, QC H3A 0C3, Canada; mahdi.chehreghani@mail.mcgill.ca; 5 Groupe de Recherche en Écologie Buccale (GREB), Faculté de Médecine Dentaire, Université Laval, Quebec City, QC G1V 0A6, Canada; 6 Félix d’Hérelle Reference Center for Bacterial Viruses, Faculté de Médecine Dentaire, Université Laval, Quebec City, QC G1V 0A6, Canada

**Keywords:** *Aeromonas salmonicida* subsp. *salmonicida*, furunculosis, phage therapy, phage cocktail, *AsaGEI*, Prophage 3

## Abstract

Aquaculture is a rapidly growing food production sector. Fish farmers are experiencing increasing problems with antibiotic resistance when fighting against pathogenic bacteria such as *Aeromonas salmonicida* subsp. *salmonicida*, the causative agent of furunculosis. Phage therapy may provide an alternative, but effective use must be determined. Here, we studied the inhibition of *A. salmonicida* subsp. *salmonicida* strains by five phages (HER98 [44RR2.8t.2], HER110 [65.2], SW69-9, L9-6 and Riv-10) used individually or as combinations of two to five phages. A particular combination of four phages (HER98 [44RR2.8t.2], SW69-9, Riv-10, and HER110 [65.2]) was found to be the most effective when used at an initial multiplicity of infection (MOI) of 1 against the *A. salmonicida* subsp. *salmonicida* strain 01-B526. The same phage cocktail is effective against other strains except those bearing a prophage (named Prophage 3), which is present in 2/3 of the strains from the province of Quebec. To confirm the impact of this prophage, we tested the effectiveness of the same cocktail on strains that were either cured or lysogenized with Prophage 3. While the parental strains were sensitive to the phage cocktail, the lysogenized ones were much less sensitive. These data indicate that the prophage content of *A. salmonicida* subsp. *salmonicida* can affect the efficacy of a cocktail of virulent phages for phage therapy purposes.

## 1. Introduction

Furunculosis is a deadly fish disease that causes heavy economic losses in salmonid farming throughout the world [1]. The causative agent of the typical form of furunculosis is *Aeromonas salmonicida* subspecies *salmonicida*, which is a psychrophilic, non-motile, Gram-negative aerobic bacilli that produces a water-soluble brown pigment on agar medium [2]. Due to the antibiotic administration in aquaculture worldwide, the number of antibiotic-resistant strains of *A. salmonicida* subsp. *salmonicida* is increasing [3]. As this species is a waterborne pathogen, the probability of horizontal gene transfer with other bacteria likely increases, turning it into a rich reservoir of antibiotic-resistant genes [4,5]. At least 15 plasmids have been found to carry a variety of antibiotic-resistant genes in *A. salmonicida* species [6].

One of the possible alternatives to treat or control bacterial infections is phage therapy [7]. Phages, also known as bacteriophages, are viral entities that specifically infect bacteria, eliminating them by cell lysis through the lytic cycle. In some cases, instead of directly killing their host bacteria similar to virulent phages, temperate ones can also integrate their genome into the bacterial chromosome and be maintained as a prophage [8]. Strains of *A. salmonicida* subsp. *salmonicida* are lysogenic as they contain many prophages and prophage-derived elements in their genomes [9,10,11,12,13]. While some of these viral sequences, such as Prophage 1 and Prophage 2, are found consistently in all strains of *A. salmonicida* subsp. *salmonicida*, others appear to be linked to the geographical origin of the strains such as Prophage 3, which is only found in 2/3 of the North American strains [9]. Interestingly, we recently isolated a phage, vB_AsaM_LPM4 (LPM4) from a deceased fish, which appears to be the infectious counterpart of Prophage 3 as both share the exact same sequence. Moreover, the temperate phage LPM4 can lysogenize naïve strains (Leduc R., pers. comm.).

Genomic islands (GEIs) called *AsaGEI*s are prophage-derived genetic elements also found in *A. salmonicida* subsp. *salmonicida* genomes. Of the six *AsaGEI*s identified so far, *AsaGEI1a* and *AsaGEI2a* are exclusively found in North American strains [9]. In addition, apart from some exceptions, *AsaGEI2a* is always found with Prophage 3 in strains of *A. salmonicida* subsp. *salmonicida* isolated from the Province of Quebec in Canada [9,14]. If they have the same integrase gene, *AsaGEIs* will always be found at the same insertion site in the chromosome of *A. salmonicida* subsp. *salmonicida*. On the other hand, *AsaGEI1* and *AsaGEI2* are integrated at distinct sites as they have different integrase genes. In a previous study, using genome sequencing and PCR genotyping, 77 strains were found to have Prophage 3 integrated into the same chromosomal site between *AsaGEI2a* and a tRNA^Leu^ gene [9]. Prophage 3 was identical in every strain analyzed at the genomic sequence level [9].

Several studies have confirmed the effectiveness of virulent phages in lysing several strains of *A. salmonicida* both in vitro [15,16,17] and in vivo [18,19,20,21] in fish infection trials using an initial multiplicity of infection (MOI) ranging from 0.01 to 10,000. Yet, these studies used only one phage with one *A. salmonicida* strain, or with a limited number of strains.

To find promising phage candidates to be used as part of a therapeutic treatment against furunculosis that affects fish farms, we previously characterized 12 phages from various origins, including three newly isolated phages from Quebec [22]. These analyses included phage clustering according to their genome and the presence/absence of integrase genes. We also determined their host range using a set 65 strains belonging to *A. salmonicida* and other species [22]. Some of these phages, including the three new isolated phages, displayed a very large host range against different subspecies of *A. salmonicida*.

Here, we investigated the efficacy of five of these phages in various cocktail combinations. Four phages (HER98 also known as 44RR2.8t.2, SW69-9, L9-6 and Riv-10) have a large host range against *A. salmonicida*. subsp. *salmonicida* while the fifth phage (HER110 also known as 65.2) has a narrower host range [22]. To take a responsible approach and to respect the 3Rs rule (Replacement, Reduction, Refinement) [23] before in vivo phage therapy experiments, we made in vitro assessment of the effectiveness of these phages against strains of *A. salmonicida* subsp. *salmonicida* with various genetic profiles but from the same geographic location (Quebec). We show that a four-phage cocktail (HER98, HER110, SW69-9 and Riv-10) used at an initial MOI of 1 was the most efficient in the tested conditions. Of note, the growth of strains carrying Prophage 3 was less inhibited by this optimized phage cocktail combination than strains without this prophage.

## 2. Materials and Methods

### 2.1. Bacterial Strains

All the *A. salmonicida* subsp. *salmonicida* strains used in this study are listed in Table 1. They were streaked on tryptic soy agar plates (TSA, Wisent, St-Bruno, QC, Canada) directly from the frozen (−80 °C) glycerol (15%) stocks. The inoculated TSA plates were incubated at 18 °C and prepared at least three days prior to their inoculation in liquid culture. The day prior to the experiment, two to three bacterial colonies per strain were inoculated in 10 mL of fresh tryptic soy broth (TSB, Wisent, St-Bruno, QC, Canada) and incubated overnight at 18 °C with agitation at 200 rpm.

### 2.2. Bacteriophages

The phages used in this study as well as some of their characteristics are presented in Table 2. Each phage was thawed from a −80 °C frozen 15% glycerol stock and approximately 100 μL of the stock was added to 10 mL of fresh TSB, which was inoculated with 1% (*v*/*v*) of a host culture of *A. salmonicida* subsp. *salmonicida* in the exponential growth phase. The cultures were incubated overnight at 18 °C with agitation at 200 rpm. Phage lysates were centrifuged for 10 min at 3200× *g* and the supernatant were filtered (0.45 µm pore size filters, Sarstedt, St-Leonard, Qc, Canada) to remove residual bacterial debris. If needed, the phage amplification was repeated one more time to increase their titers. To know the exact phage titer, 10-fold serial dilutions (up to 10^−7^) of each phage lysate were first made in buffer (50 mM Tris-HCl pH 7.5, 100 mM NaCl, 8 mM MgSO_4_). Then, 200 μL of the overnight culture of the bacterial host and 100 μL of each phage dilution were added to 3 mL of TSA soft agar (0.75%) kept at 55 °C and poured onto a TSA plate. Plates were incubated at 18 °C overnight and titers were calculated according to the number of plaques obtained and dilution used.

### 2.3. Growth Curves of A. salmonicida subsp. salmonicida Strains in Presence of Phages

Bacterial cultures (Table 1) in the exponential growth phase and phages (Table 2) were incubated in a sterile 48-well microplate with a flat bottom (Corning, Toronto, On, Canada) at different MOIs and phage combinations in a final volume of 300 μL. The phage combinations had an equal mix of each phage (Table S1). The optical density (OD) at 595 nm was adjusted to 0.1. The experiments were all performed using a microplate reader (Infinite 200 PRO, Tecan, Baldwin Park, CA, USA), with continuous shaking with 3.5 mm amplitude in orbital mode at 200 rpm and readings performed every 15 min at 19 °C for 4 days. All the experiments were performed at three initial MOIs (0.1, 1, and 10) in triplicates or more.

### 2.4. Data Analysis

**Figure 1:**
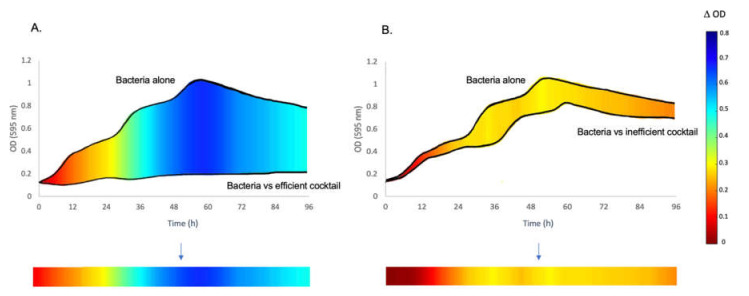
Example of the schematic representation of the ∆OD values of bacterial growth with or without phages. To simplify the presentation of the results for the large number of conditions tested, a schematic representation was developed to show the phage effectiveness in inhibiting bacterial growth. (**A**). The two growth curves show the OD of the bacterial strain grown alone and in the presence of the best phage cocktail. The colored line below the graph represents the ∆OD of the growth curve of bacteria alone minus the one of bacteria in the presence of phages based on the range shown on the right of the figure. (**B**). Same principle as in panel A, but the bacteria were in the presence of phages that were not effective growth inhibitors. When the bacterial growth is highly inhibited, the color of the line tends to blue. No inhibition corresponds to red lines.

Data analysis was performed using standard Excel functionality to calculate the average and ∆OD values (Figure 1). Subsequently, the Excel sheets obtained were processed with Matlab R2020a (MathWorks, Inc., Natick, MA, USA) to create the colored line representations of the phage effect on bacterial growth. The results are represented by a line for each condition with a color code; the growth difference between the bacteria without treatment and the bacteria in the presence of phages is illustrated (Figure 1). Specifically, all the values used to design the colored lines are the differences of the OD values at 595 nm between the bacterial strain grown alone or in the presence of phages. The spectrum of colors begins with red, representing the ∆OD of zero, and the values increase (yellow = ∆OD 0.3 and green = ∆OD 0.4 − 0.5) by moving towards the dark blue color, representing the maximum ∆OD values and inhibition of growth (ending at 0.8).

The local Virulence Index (Vi) of the various conditions tested was also evaluated as previously described [29]. Vi varies within a range from 0 to 1 where 1 represents the maximal virulence of phage(s).

## 3. Results and Discussion

### 3.1. The Inhibitory Effect of Phage Combinations on Bacterial Growth

The purpose of this study was to determine which phage combinations will lead to the maximum inhibition of bacterial growth in liquid medium. The model bacterial strain chosen for this initial evaluation was *A. salmonicida* subsp. *salmonicida* 01-B526. This strain was isolated in the Province of Quebec, possesses an *AsaGEI1a* and six plasmids (pAsa1, pAsa2, pAsa3, pAsal1, pAsa5, pAsa9) but no Prophage 3 [9,27,30]. The inhibitory effect of five individual phages and of 24 different combinations (cocktails A to X) of these phages on strain 01-B526 was determined using initial MOIs of 0.1, 1, and 10. See Supplementary Data Table S1 for the description of the phage combinations.

**Figure 2:**
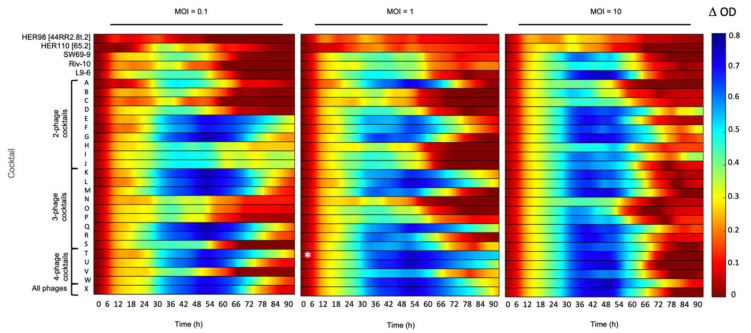
Growth inhibition of strain 01-B526 in presence of various phage combinations. The effect of individual phages and 24 phage combinations (A to X, see Table S1 for phage combination details) at three different MOIs (0.1, 1, and 10) on the growth of strain 01-B526 are shown from left to right. The colored lines representing the growth inhibition by phages have been determined as shown in Figure 1. When the bacterial growth is highly inhibited, the color of the line will tend to blue. No inhibition corresponds to red lines. According to the colored lines, Cocktail T, which included phages HER98 + HER110 + SW69-9 + Riv-10 (MOI = 1), is the best combination for bacterial growth inhibition (white asterisk).

The individual phages (HER98 [44RR2.8t.2], HER110 [65.2], SW69-9, Riv-10, and L9-6) generally inhibited bacterial growth less than most of the phage cocktails (Figure 2). From an MOI perspective, starting with an MOI of 10 had in general a higher inhibition than lower MOI values, but the effect did not last over time. After 66 h of incubation at an initial MOI of 10, the bacterial culture seems to have recovered from the initial phage infections as the ODs increased.

The least inhibition of bacterial growth was observable with phage HER98 [44RR2.8t.2], isolated in Ontario (Canada), and at the three MOIs tested. This weak inhibition of bacterial growth was also observed with the two or three-phage cocktails containing this phage as well as those isolated in Quebec (SW69-9, Riv-10, L9-6) at MOIs 0.1 and 1 (Combinations B, C, D, N, O, and P, Table S1). The ineffectiveness of Quebec-isolated phages can also be clearly tracked by focusing on cocktail S containing phages SW69-9, Riv-10, and L9-6 at MOIs 0.1 and 1. This S cocktail was a less effective inhibitor against strain 01-B526, which is only comparable with three-part cocktails N, O, and P. Therefore, it seems that the combinations of the phages used in this study and isolated in Canada (HER98 [44RR2.8t.2], SW69-9, Riv-10, and L9-6) were not efficient in preventing the growth of the strain 01-B526 isolated in Quebec.

Phage HER110 [65.2], isolated from the Saône-et-Loire River in France, seemed to be a better candidate for two or three-phage cocktails (E, F, G, Q and R, Table S1) as the bacterial growth was strongly inhibited (∆OD_595nm_ > 0.6) even after 72 h of incubation at all three MOIs tested (Figure 2). Strain 01-B526 was also strongly inhibited when in presence of the cocktail W, containing four phages HER110 [65.2], SW69-9, Riv-10, and L9-6, at the three MOIs used. These results suggest that phages isolated from the same geographical region as the targeted strain may not necessarily be the best adapted and suitable to inhibit bacterial growth.

It could therefore be interesting, within the limits of biosafety constraints imposed by government agencies, to introduce phages from another region for the treatment of furunculosis in a specific region. It has been already demonstrated to control the growth of *Sphingomonas* sp. with phages [31]. In fact, using phages from another region has been proposed as a general rule to mitigate low phage activity [32].

Except for the cocktails containing both phages HER98 [44RR2.8t.2] and HER110 [65.2], strain 01-B526 showed growth (especially after 66 h) in presence of the other phages in various combinations and initial MOIs. For most of the conditions tested, bacterial growth even appeared after 30 h. In a similar study, early growth of *A. salmonicida* in presence of phages was reported as early as 2.5 h after the beginning of the phage–bacteria incubation. Both bacteria and phages had been isolated from China [15].

Finally, the best phage cocktail in reducing the growth of 01-B526 was the combination of the four phages HER98 [44RR2.8t.2], HER110 [65.2], SW69-9 and Riv-10 (Cocktail T) (Figure 2). This combination was the most effective cocktail in preventing bacterial growth at initial MOI of 1 since the ∆OD was never lower than 0.5, even after almost 4 days. Moreover, this cocktail displayed the highest Vi value (Table S2).

### 3.2. Effect of the Best Phage Cocktail on Various Strains with Different Genetic Backgrounds

As cocktail T, including four phages, was identified as the most effective in inhibiting the growth of strain 01-B526, we tested its efficacy against other *A. salmonicida* subsp. *salmonicida* strains with different genetic profiles (Table 1). Five different groups of bacterial strains were tested. Group I included two strains from Europe without *AsaGEI* or Prophage 3. All the other groups included strains from Quebec (except one from New Brunswick, a neighboring province of Quebec). Group II strains contained no *AsaGEI* or Prophage 3, Group III strains had only an *AsaGEI1a,* Group IV strains had only an *AsaGEI2a*, and Group V strains bear both *AsaGEI2a* and Prophage 3.

**Figure 3:**
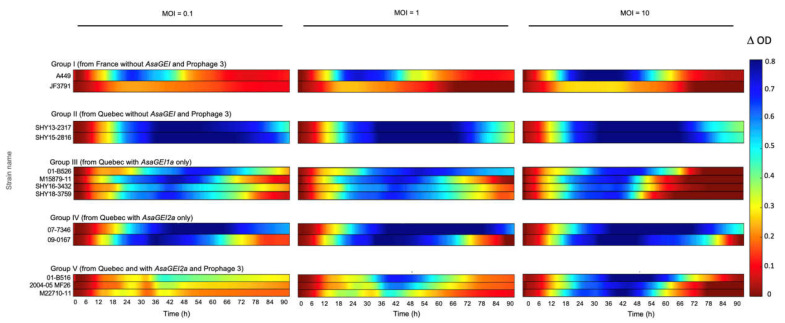
Effect of the phage cocktail T on the growth of various *A. salmonicida* subsp. *salmonicida* strains with different genetic backgrounds. Bacteria were incubated with the phage cocktail at different initial MOIs (0.1, 1, and 10) from left to right. The colored lines representing the growth inhibition by phages have been deduced as shown in Figure 1. When the bacterial growth is highly inhibited, the color of the line will tend to blue. No inhibition corresponds to red lines. The bacterial growths with the phage cocktail at MOI of 10 are especially obvious for strains in Groups I, III, and V after 54 h. In addition, bacterial growth is noticeable for Group V during the exponential phase approximately around 30 h for MOIs 0.1 and 1.

The effectiveness of the phage cocktail varied between groups (Figure 3). Among Group I strains, JF3791 showed lower ODs in general than the other European strain A449 (Supplementary data, Figure S1), which translated into graphical data that were seemingly redder in comparison to A449 (Figure 3, Group I). Our graphical representation method gave the impression that the phages had a lesser effect, which was not the case. Indeed, growth inhibition was similar to strain A449, according to the growth curves (Figure S1). These results show that our graphical representation method is more adequate when strains have an equivalent level of growth. This is the case for all the strains studied here except JF3791.

For strains belonging to groups II to V, the phage cocktail was especially less efficient when tested on strains containing Prophage 3 (Group V) at MOIs 0.1 and 1 (Figure 3 and Table S3). It was shown previously [9] that Prophage 3 is extensively present in North American strains that also bear *AsaGEI2a*, except for a few strains [14]. Group IV consists of strains that contain *AsaGEI2a* but without Prophage 3, and they were clearly sensitive to the phage cocktail. The specific role of Prophage 3 is highlighted especially when Groups IV and V are compared, clearly showing reduced sensitivity to the virulent phage cocktail, especially at lower MOIs (Figure 3).

**Figure 4:**
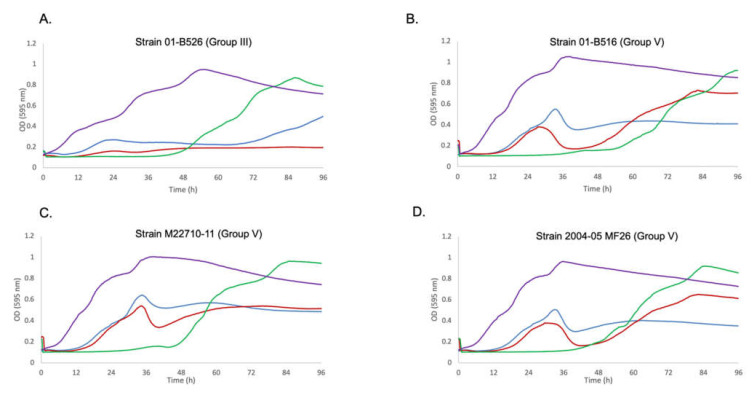
Growth curves for Group III and V strains against Cocktail T. (**A**). Growth curve of strain 01-B526 (Group III) is shown. The purple curve is the bacterium alone. The blue, red and green curves are the growth of the bacterial strain with Cocktail T at MOIs 0.1, 1, and 10, respectively. The same color code of the curves applies to all panels of this figure. Here, the highest bacteria growth inhibition is for MOI 1. Bacterial growth can be seen for MOI 10 after 48 h. (**B**–**D**). Growth curves of strains 01-B516, M22710-11, and 2004-05 MF26, respectively (Group V). These strains bear *AsaGEI2a* and Prophage 3. The ineffectiveness of the cocktail due to the presence of Prophage 3 can be observed during the logarithmic phase in case of MOIs 0.1 and 1.

Overall, the bacterial growth inhibition phenotype with different MOIs was a strain-dependent phenomenon. For strains from Quebec with no specific genetic element (Group II), growth inhibition was observed at all MOIs. Group III having *AsaGEI1a*, had a strong growth in presence of phages at an initial MOI of 10, especially noticeable after 48 h while lower MOIs led to more efficient growth inhibition. Strains belonging to Group IV (*AsaGEI2a*) were inhibited by phages at various MOIs. For Group V (*AsaGEI2a* and Prophage 3), a different trend was observed. At higher MOI, the growth inhibition improved, but almost all the strains recovered after 66 h. The Group III and V strains had similar behavior at MOI 10 (Figure 3 and Figure 4).

Finally, apart from GEIs and Prophage 3, some of the strains analyzed in this part of the study contained different plasmids (pAsa4, pSN254b, pRAS3-like) [4,5,33], carrying genes coding for resistance to antibiotics. None of these plasmids were found to have an impact on the sensitivity of bacterial strains to the phage cocktail.

### 3.3. Confirmation of the Role of Prophage 3 in Reducing the Effectiveness of Phage Growth Inhibition

Since Group V strains contain both *AsaGEI2a* and Prophage 3, we could not exclude that the inhibitory effect on phage activity could be due to a combined action of both genetic elements. We recently isolated the temperate phage LPM4, which is identical to Prophage 3. The infectious particle LPM4 can be used to lysogenize strains lacking Prophage 3 including strains from Group I to IV of the present study (Leduc G., pers. comm.), thereby confirming the inhibitory role of Prophage 3 on the phage cocktail effectiveness (Table 1).

**Figure 5:**
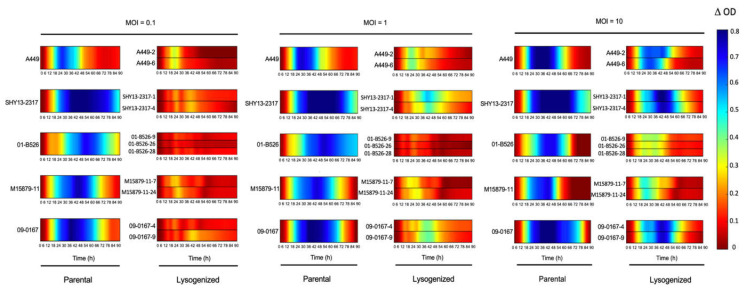
Confirmation of the role of Prophage 3 on the effectiveness of the phage Cocktail T. The comparison of the efficacy of the phage cocktail T on parental strains without Prophage 3 and the lysogenized strains containing Prophage 3 are shown at three different MOIs. For each MOI, the parental strains are illustrated in the first column, and the second column contains the strains lysogenized with Prophage 3. The colored lines representing the growth inhibition by phages have been deduced as shown in Figure 1. When the bacterial growth is highly inhibited, the color of the line will tend to blue. No inhibition corresponds to red lines.

This was clearly obvious by both the graphical representation (Figure 5) and the Vi values (Table S4). Prophages have been shown previously to play significant roles in bacterial fitness and evolution, such as: (1) increasing the fitness of bacteria through the lysogenic conversion process, or (2) providing phage resistance through superinfection exclusion [34,35,36]. Examples of immunity through the presence of prophage (myophages 43-10T and 1-16T) has even been shown with *A. salmonicida* subsp. *salmonicida* [37,38]. The system (and its mode of action) responsible for the phage protection provided by the prophages 43-10T and 1-16T has yet to be explored. Further molecular studies are also required to confirm how Prophage 3 acts to protect against this phage cocktail. Nonetheless, the protection provided by Prophage 3 against virulent phages is likely one of the reasons why two-thirds of the *A. salmonicida* subsp. *salmonicida* strains from Quebec carry this prophage [9]. It also highlights the need to isolate new virulent phages capable of lysing strains with the Prophage 3 to improve the efficacy of in vivo phage treatments.

We previously studied the host range of these virulent phages using spot assays on a solid medium [22] and we did not observe any particular pattern regarding strains carrying or not Prophage 3 as observed in liquid medium. We redid the host range analysis of these phages on plates with the lysates used in this study and we still did not observe any pattern related to Prophage 3 (Table S5). For example, the strain 01-B516 with a Prophage 3 was as sensitive as the other strains tested with all the phages, except for phage L9-6 which was not used in the cocktail T. Clearly, the liquid and solid media assays offer different environmental conditions to study phage–bacteria interactions. In vivo studies in fish are needed to provide additional data to help determine which in vitro assays is more representative for phage therapy purposes in aquaculture.

## 4. Conclusions

To devise an efficient in vivo phage therapy treatment, phage–bacteria interaction must first be studied in vitro. Here, we confirmed that cocktails of phages function better than individual phages to inhibit the growth of a strain responsible for furunculosis in Quebec. As phage–bacteria interactions are dynamic, the regional context of bacterial strains may be a factor in selecting phage candidates against furunculosis. A next step would be to try cocktails containing phages from other parts of the world, since we show that the combination of a phage isolated in France can provide additional inhibitory effect. This study also clearly demonstrated the negative effect of Prophage 3 on the potential treatment of furunculosis and the isolation of new phages should be performed using such strain as a host. Finally, while the in vitro experiments were justified regarding the 3Rs rule by reducing the number of conditions tested, fish studies are still needed to validate the in vitro data, particularly to help predicting the efficacy of a given phage cocktail in aquaculture.

## Figures and Tables

**Table 1:** *A. salmonicida* subsp. *salmonicida* strains used in this study.

Strain	Characteristic	Origin	Reference ^a^	Group ^b^	*AsaGEI* ^c^	Pro. 3 ^d^	TTSS ^e^	A-Layer	ARG ^f^
A449	Virulent	Fish infection, France	[12]	I	No	No	Yes	Yes	*sul1, cat, tetA(E)*
JF3791	Virulent	Fish infection, Switzerland	[24]	I	No	No	No	Yes	No
SHY13-2317	Virulent	Fish infection, Quebec, Canada	[25]	II	No	No	Yes	Yes	No
SHY15-2816	Virulent	Fish infection, Quebec, Canada	[26]	II	No	No	Yes	Yes	No
01-B526	Virulent	Fish infection, Quebec, Canada	[27]	III	*1a*	No	Yes	Yes	No
M15879-11	Virulent	Fish infection, Quebec, Canada	[28]	III	*1a*	No	Yes	Yes	*sul1, sul2, floR, tetA*
SHY16-3432	Virulent	Fish infection, Quebec, Canada	[4]	III	*1a*	No	Yes	Yes	*sul1, sul2, floR, tetA*
SHY18-3759	Virulent	Fish infection, Quebec, Canada	[14]	III	*1a*	No	Yes	Yes	*tetA(C)*
07-7346	Virulent	Fish infection, Quebec, Canada	[25]	IV	*2a*	No	Yes	Yes	No
09-0167	Virulent	Fish infection, Quebec, Canada	[25]	IV	*2a*	No	Yes	Yes	No
01-B516	Virulent	Fish infection, Quebec, Canada	[25]	V	*2a*	Yes	Yes	Yes	No
2004-05 MF26	Virulent	Fish infection, New-Brunswick, Canada	[25]	V	*2a*	Yes	Yes	Yes	*sul1, sul2, floR, tetA*
M22710-11	Virulent	Fish infection, Quebec, Canada	[25]	V	*2a*	Yes	Yes	Yes	No
HER1098	Phage host	Brook trout, USA	FHRC	N/A	No	No	No	No	No
HER1110	Phage host	Trout, Japan	FHRC	N/A	No	No	No	No	No
AS-R5	Phage host	Rearranged strain from m15879-11	[28]	N/A	*1a*	No	No	No	*sul1, sul2, floR, tetA*
AS-19-R1	Phage host	Rearranged strain from 08-2783	This study and [14]	N/A	*1a*	No	No	Yes	*sul1, sul2, floR, tetA*

^a^ FHRC: Felix d’Herelle Reference Center for Bacterial Viruses. ^b^ N/A: Not applicable. ^c^ *AsaGEI*: *Aeromonas salmonicida* GEnomic Island. ^d^ Pro. 3: Prophage 3. ^e^ TTSS: Type three secretion system, major virulence factor of *Aeromonas salmonicida.* ^f^ ARG: Antibiotic resistance genes: *su11* and *sul2*; sulfonamide resistance, *cat* and *floR;* chloramphenicol resistance, *tetA, tetA*(C) and *tetA*(E)*:* tetracycline resistance.

**Table 2:** Bacteriophages infecting *A. salmonicida* subsp. *salmonicida* used in this study.

Bacteriophage	Family (Genus)	Genome Size (bp)	GenBank	Origin	Host Strain	Reference ^a^
HER98 (44RR2.8t.2) ^b^	*Myoviridae* (*Biquartavirus)*	173,590	KY290948	Water, Ontario, Canada	HER1098	FHRC
HER110 (65.2) ^b^	*Myoviridae*	236,567	KY290955	River, Saône-et-Loire, France	HER1110	FHRC
L9-6	*Myoviridae*	173,578	KY290956	Lake, Quebec, Canada	AS-19-R1	[22]
Riv-10	*Myoviridae*	174,311	KY290957	River, Quebec, Canada	AS-R5	[22]
SW69-9	*Myoviridae*	173,097	KY290958	Source, Quebec, Canada	AS-R5	[22]

^a^ FHRC: Felix d’Herelle Reference Center for Bacterial Viruses. ^b^ The alternative name of this phage is shown in parentheses.

## Data Availability

Data are contained within the article and Supplementary Material. The raw data of this study are available on request from the corresponding author.

## Supplementary Materials

The following are available online at https://www.mdpi.com/article/10.3390/v13112241/s1, Table S1. Phage cocktails used in this study. Table S2. Local virulence index for various phage combinations against *Aeromonas salmonicida* subsp. *salmonicida* strain 01-B526. Table S3. Local virulence index for cocktail T against various *A. salmonicida* subsp. *salmonicida* strains with different genetic backgrounds. Table S4. Local virulence index for cocktail T against different *A. salmonicida* subsp. *salmonicida* strains with or without Prophage 3. Table S5. Sensitivity of *A. salmonicida* subsp. *salmonicida* strains harboring various genetic elements to five individual phages using the spot test assay. Figure S1. Growth curves for the European strains A449 and JF3791 in presence of the phage cocktail T (MOIs 0.1, 1 or 10) compared to the growth of the strains without phages.
