# Supplementary material for: Phage Cocktail Development against Aeromonas salmonicida subsp. salmonicida Strains Is Compromised by a Prophage
Source: Viruses. 2021 Nov 8;13(11):2241. doi: 10.3390/v13112241 (PMC8621227; doi:10.3390/v13112241)
Supplement: Supplementary file 1 [file viruses-13-02241-s001.zip › viruses-1431545-supplementary.pdf]

viruses

Supplementary data

Phage Cocktail Development Against *Aeromonas salmonicida* subsp. *salmonicida* Strains is Compromised by a Prophage

Nava Hosseini [1,2,3], Valérie E. Paquet [1,2,3], Mahdi Chehreghani [4], Sylvain Moineau [1,2,5,6] and Steve J. Charette [1,2,3,*]

[1] Institut de Biologie Intégrative et des Systèmes (IBIS), Pavillon Charles-Eugène-Marchand, Université Laval, Quebec City, QC G1V 0A6, Canada; nava.hosseini.1@ulaval.ca (N.H.); Valerie.Paquet@criucpq.ulaval.ca (V.E.P.); Sylvain.Moineau@bcm.ulaval.ca (S.M.)
[2] Département de Biochimie, de Microbiologie et de Bio-Informatique, Faculté des Sciences et de Génie, Université Laval, Quebec City, QC G1V 0A6, Canada
[3] Centre de Recherche de l'Institut Universitaire de Cardiologie et de Pneumologie de Québec (IUCPQ), Quebec City, QC G1V 4G5, Canada
[4] Department of Mechanical Engineering, McGill University, Montreal, QC H3A 0C3, Canada; mahdi.chehreghani@mail.mcgill.ca
[5] Groupe de Recherche en Écologie Buccale (GREB), Faculté de Médecine Dentaire, Université Laval, Quebec City, QC G1V 0A6, Canada
[6] Félix d'Hérelle Reference Center for Bacterial Viruses, Faculté de Médecine Dentaire, Université Laval, Quebec City, QC G1V 0A6, Canada
* Correspondence: steve.charette@bcm.ulaval.ca

Table S1. Phage cocktails used in this study.

Identification of phage cocktails	Combination of virulent phages	Proportion (%)
A	HER98 + HER110	50–50
B	HER98 + SW69-9	50–50
C	HER98 + RIV-10	50–50
D	HER98 + L9-6	50–50
E	HER110 + SW69-9	50–50
F	HER110 + RIV-10	50–50
G	HER110 + L9-6	50–50
H	SW69-9 + RIV-10	50–50
I	SW69-9 + L9-6	50–50
J	RIV-10 + L9-6	50–50
K	HER98 + HER110 + SW69-9	33 –33 –33
L	HER98 + HER110 + RIV-10	33 –33 –33
M	HER98 + HER110 + L9-6	33 –33 –33
N	HER98 + SW69-9 + RIV-10	33 –33 –33
O	HER98 + SW69-9 + L9-6	33 –33 –33
P	HER98 + RIV-10 + L9-6	33 –33 –33
Q	HER110 +SW69-9 + RIV-10	33 –33 –33
R	HER110 +SW69-9 + LP9-6	33 –33 –33
S	SW69-9 + RIV-10 + L9-6	33 –33 –33
T	HER98 + HER110 + SW69-9 + RIV-10	25 –25 –25 –25
U	HER98 + HER110 + SW69-9 + L9-6	25 –25 –25 –25
V	HER98+ SW69-9 + RIV-10 + L9-6	25 –25 –25 –25
W	HER110 + SW69-9 + RIV-10 + L9-6	25 –25 –25 –25
X	HER98+ HER110 + SW69-9 + RIV-10 + L9-6	20–20–20–20–20

Table S2. Local virulence index for various phage combinations against Aeromonas salmonicida subsp. salmonicida strain 01-B526. The highest value is indicated in blue.

Phage cocktail	vi_MOI 0.1	vi_ MOI 1	vi_ MOI 10
HER98 [44RR2.8t.2]	0.046044454	0.186651001	0.218289561
HER110 [65.2]	0.199182396	0.189452846	0.185404235
SW69-9	0.277307759	0.37496482	0.408130133
Riv-10	0.16298522	0.385106676	0.398548002
L9-6	0.301305524	0.364086701	0.528888694
A	0.322921671	0.538446462	0.243112231
B	0.255301344	0.223085183	0.350845633
C	0.155787975	0.199480607	0.378664711
D	0.312894356	0.336540973	0.618233469
E	0.677996348	0.5522271	0.504084348
F	0.633118652	0.569655485	0.610741481
G	0.657725414	0.506799038	0.62548578
H	0.465167133	0.239655633	0.443879947
I	0.517659676	0.300406537	0.575425561
J	0.518535142	0.296989504	0.445726203
K	0.670512383	0.62578379	0.496826045
L	0.639308411	0.685903562	0.493491761
M	0.633062893	0.494415756	0.52940613
N	0.321471574	0.180811498	0.329640898
O	0.329127671	0.317298372	0.358358222
P	0.279562852	0.389207927	0.437787149
Q	0.686963291	0.685108518	0.58524618
R	0.640020707	0.466719635	0.526446416
S	0.278722943	0.546341345	0.503507984
T	0.628657638	0.755075302	0.483464445
U	0.567288228	0.740415831	0.458657078
V	0.274923864	0.518826269	0.479194182
W	0.619498904	0.694638949	0.619035693
X	0.578853834	0.704268718	0.513593931

Table S3. Local virulence index for cocktail T against various A. salmonicida subsp. salmonicida strains with different genetic backgrounds.

Strain types	vi_MOI 0.1	vi_ MOI 1	vi_ MOI 10
**Cocktail T, local virulence index against European strains, without *AsaGEI* and Prophage 3**			
A449	0.336424818	0.40274039	0.488505662
JF379	0.448689918	0.282736617	0.410928338
**Cocktail T, local virulence index against Quebec strains, without *AsaGEI* and Prophage 3**			
SHY13-2317	0.717108707	0.733791365	0.745477031
SHY15-2816	0.749678939	0.72496142	0.757712432
**Cocktail T, local virulence index against Quebec strains, with *AsaGEI1a***			
01-B526	0.628657638	0.755075302	0.483464445
M15879-11	0.624797154	0.612398984	0.396309815
SHY16-3432	0.603905802	0.635290645	0.424066315
SHY18-3759	0.601946397	0.663228334	0.385872447
**Cocktail T, local virulence index against Quebec strains, with *AsaGEI2a***			
07-7346	0.701869888	0.786060585	0.766785491
09-0167	0.593843682	0.626626347	0.709431381
**Cocktail T, local virulence index against Quebec strains, with *AsaGEI2a* and Prophage 3**			
01-B516	0.396935099	0.463445471	0.594729104
2004-05MF26	0.336003899	0.436750735	0.48265839
M22710-11	0.27512771	0.318053256	0.461495858

Table S4. Local virulence index for cocktail T against different A. salmonicida subsp. salmonicida strains with or without Prophage 3**.**

Strain types	vi_MOI 0.1	vi_ MOI 1	vi_ MOI 10
A449 (-Pro3)	0.494013072	0.406029264	0.494013072
A449-2 (+Pro3)	0.10437813	0.242622392	0.486418874
A449-6 (+Pro3)	0.150866628	0.224389906	0.377181179
SHY13-2317 (-Pro3)	0.720299269	0.738424194	0.749839586
SHY13-2317-1 (+Pro3)	0.183734639	0.392304956	0.538994321
SHY13-2317-4 (+Pro3)	0.178533094	0.320080035	0.575236751
01-B526 (-Pro3)	0.632215908	0.755345052	0.490093747
01-B526-9 (+Pro3)	0.151169151	0.14899264	0.367778442
01-B526-26 (+Pro3)	0.102226017	0.102393423	0.329766714
01-B526-28 (+Pro3)	0.148080043	0.115742069	0.317723029
M15879-11 (-Pro3)	0.629882183	0.618436844	0.402461793
M15879-11-7 (+Pro3)	0.17513866	0.177666145	0.30819099
M15879-11-24 (+Pro3)	0.189023558	0.225972272	0.304579525
09-0167 (-Pro3)	0.598024326	0.634030166	0.717258134
09-0167-4 (+Pro3)	0.146702951	0.325207204	0.567615586
09-0167-9 (+Pro3)	0.223901283	0.312552123	0.513292927

Table S5. Sensitivity of *A. salmonicida* subsp. *salmonicida* strains harboring various genetic elements to five individual phages using the spot test assay.

Strain	Phages				
	**HER98**	**HER110**	**SW69-9**	**RIV-10**	**L9-6**
M15879-11 (*AsaGEI1a*)	10^{-4}-10^{-7}	10^{-1}-10^{-3}	10^{-4}-10^{-7}	10^{-4}-10^{-7}	10^{-4}-10^{-7}
SHY13-2317 (no *AsaGEI* or Prophage 3)	10^{-4}-10^{-7}	10^{-1}-10^{-3}	10^{-4}-10^{-7}	10^{-4}-10^{-7}	10^{-4}-10^{-7}
01-B516 (*AsaGEI2a* + Prophage 3)	10^{-4}-10^{-7}	10^{-1}-10^{-3}	10^{-4}-10^{-7}	10^{-4}-10^{-7}	10^{-1}-10^{-3}

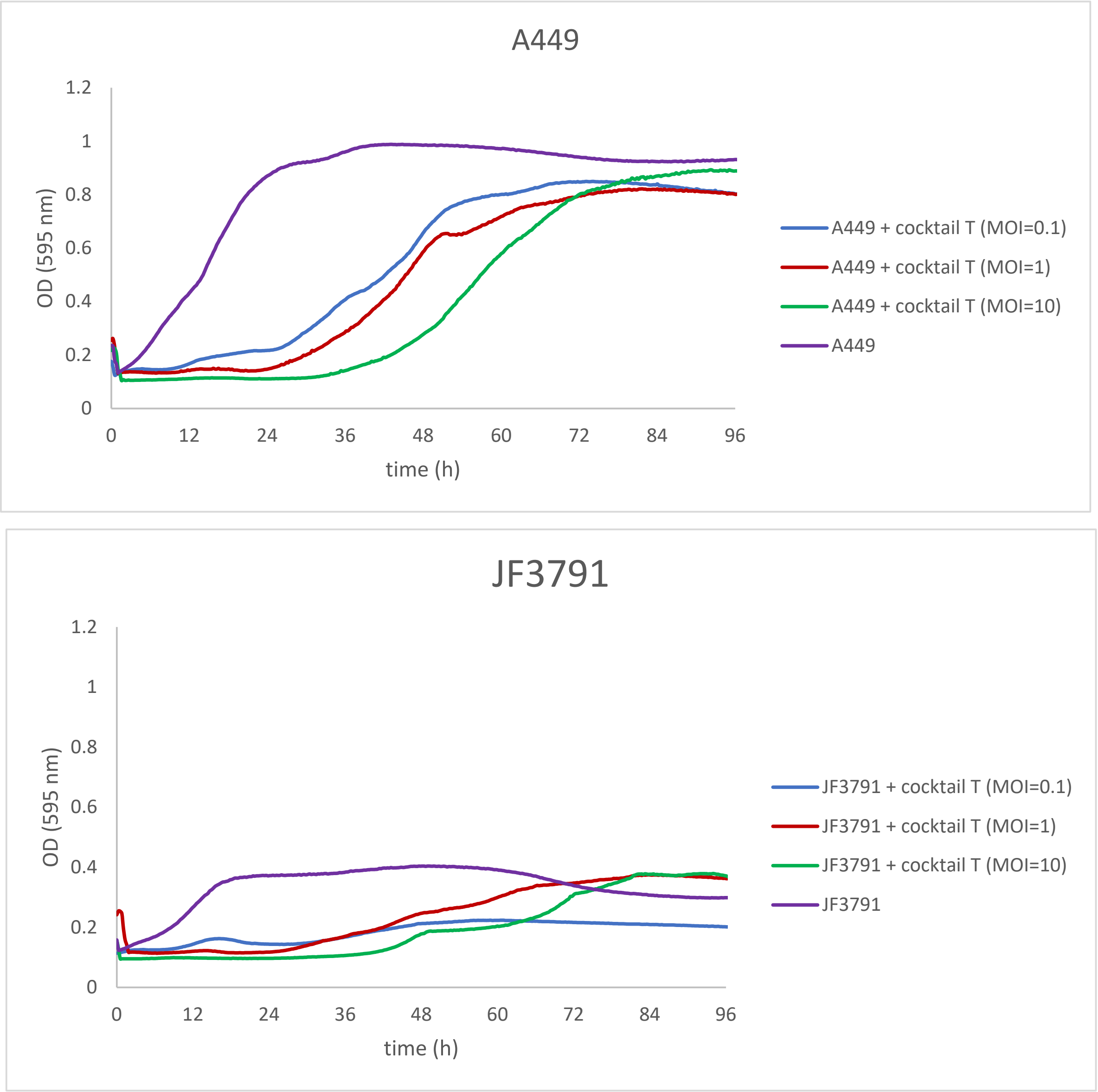

Figure S1. Growth curves for the European *A. salmonicida* subsp. *salmonicida* strains A449 and JF3791 in presence of the phage cocktail T (at MOIs 0.1, 1 or 10) compared to the growth of the bacterial strains without phages. The curves are the average of three biological replicates.
